# *Earliella scabrosa*-associated postoperative Endophthalmitis after Phacoemulsification with intraocular lens implantation: a case report

**DOI:** 10.1186/s12886-018-0702-9

**Published:** 2018-02-17

**Authors:** Hong He, Xiaolian Chen, Hongshan Liu, Jiaochan Wu, Xingwu Zhong

**Affiliations:** 1 Hainan Eye Hospital and Key Laboratory of Ophthalmology, Zhongshan Ophthalmic Center, Sun Yat-sen University, 19 Xiuhua Road, Haikou, China; 2 Zhongshan Ophthalmic Center and State Key Laboratory of Ophthalmology, Sun Yat-sen University, Guangzhou, China

**Keywords:** Postoperative Endophthalmitis, *Earliella scabrosa*, Ocular fungal infection

## Abstract

**Background:**

Postoperative endophthalmitis after cataract surgery is a severe eye infection that can lead to irreversible blindness in the affected eye. The characteristics, treatment and prognosis of this disease vary because of its association with different pathogens. Here, we report what is possibly the first case of endophthalmitis after cataract surgery to be associated with the rare pathogen *Earliella scabrosa*.

**Case presentation:**

A 56-year-old man from Hainan Island (China) with a history of phacoemulsification and type II diabetes mellitus underwent intraocular lens (IOL) implantation. He later presented with progressive endophthalmitis in his right eye. IOL explantation with capsular bag removal and a 23G pars plana vitrectomy combined with a silicone oil tamponade was performed. The infection was cleared without recurrence, and the patient’s visual acuity improved from light perception to 20/200 in the right eye. An in vitro culture determined that the causative pathogen was *Earliella scabrosa*, and this result was confirmed by an internal transcribed spacer (ITS) sequence analysis.

**Conclusion:**

*Earliella scabrosa* has never been reported as an infectious agent in human eyes, and its clinical significance remains unknown. Here, we report a rare case of *Earliella scabrosa*-associated endophthalmitis after cataract surgery. The fungal infection presented as an acute attack and was successfully treated with vitrectomy.

## Background

Postoperative endophthalmitis is one of the most severe complications of cataract surgery and can result in extremely poor vision. However, its causative pathogens vary among different regions. Different fungi have been identified as a prime causative agents in developing countries with tropical and subtropical climates. For example, Anand et al. demonstrated that fungi accounted for 21.8% of 170 eyes with postoperative endophthalmitis in southern India [1]. Another large case series from India involved 124 eyes and revealed that over half of the cases involved a fungal infection [2]. The spectrum of fungi described in previous studies includes *Aspergillus* spp., *Candida* spp., *Acremonium falciforme*, *Paecilomyces* spp., *Fusarium* spp., and *Curvularia* spp. To the best of our knowledge, ours is the first reported case of endophthalmitis after cataract surgery to be associated with the rare pathogen *Earliella scabrosa*.

## Case presentation

**Fig. 1:**
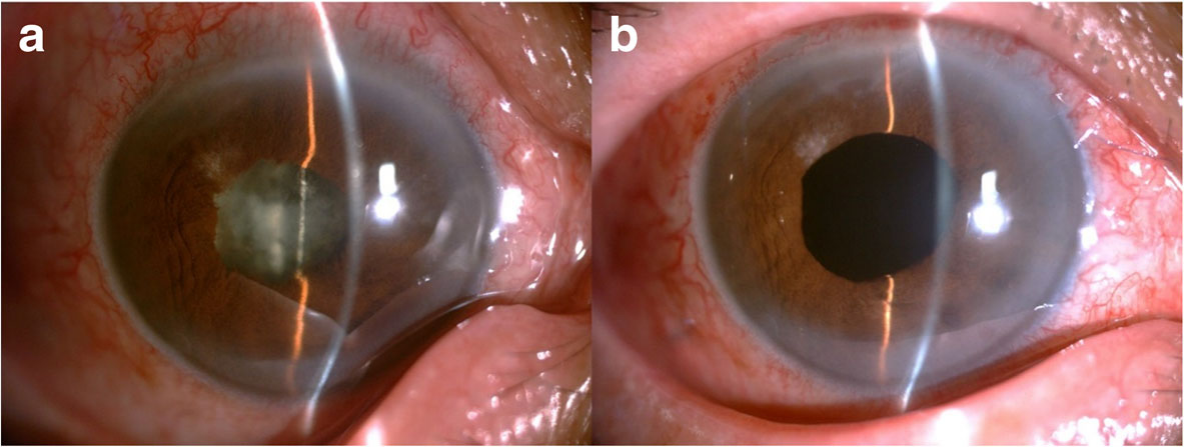
Photographs of the infected ocular area. **a** The right eye (with the IOL) showed severe vitritis with no fundus view. **b** No recurrence was observed at 6 months after the IOL explantation was performed with capsular bag removal and 23G pars plana vitrectomy combined with a silicone oil tamponade

A 56-year-old male patient with a history of type II diabetes mellitus was referred to the Hainan Eye Hospital (Haikou, China) for a red and painful right eye with poor vision. One month before admission, the patient underwent phacoemulsification and IOL implantation in a local hospital. Within 72 h of this surgery, he presented at a private clinic with irritation, redness and reduced vision in the right eye. The patient was treated with antibacterial medications (levofloxacin eye drops, six times per day) for 3 days, and it was suggested that he present for a subsequent visit 3 days later. However, the patient missed this follow-up visit, and he applied the levofloxacin eye drops for 1 month. At 1 month after his presentation at the private clinic, his signs and symptoms had not improved. It was at this time that the patient was referred to the Hainan Eye Hospital (Haikou, China). Upon arrival, his visual acuity was light perception in the right eye and 20/20 in the left eye. A slit-lamp examination revealed conjunctival injection, a positive Tyndall effect (+) in the anterior chamber, and severe vitritis with no fundus view (Fig. 1a). Acute post-cataract endophthalmitis was suspected.

IOL explantation with capsular bag removal and a 23G pars plana vitrectomy combined with a silicone oil tamponade was performed. The intraocular irrigation solution used during surgery contained 1 mg/0.1 ml vancomycin to treat a possible bacterial infection. In addition, a vitreous biopsy was obtained for culture. Fortified tobramycin and levofloxacin eye drops were started after the vitrectomy and continued for 6 days. One week after surgery, the patient achieved a best-corrected visual acuity of 20/200. The intraocular silicon oil was removed after 6 months, and no recurrence was observed (Fig. 1b).

**Fig. 2:**
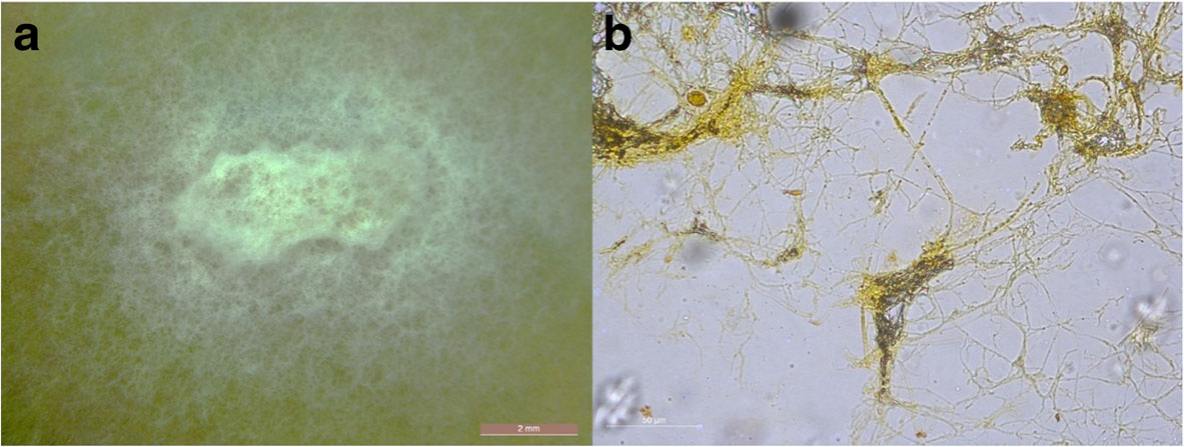
The morphological characteristics of *Earliella scabrosa*. **a** The pathogen isolated from the patient was cultured in SDA and formed a white colony. **b** The hyphae stained positive for acridine orange, and the results indicated an extremely thick cell wall, sparse septae, and internal nuclei (200×)

**Fig. 3:**
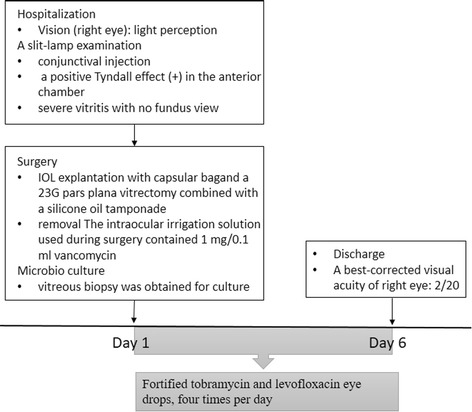
Timeline of interventions and outcomes

The vitreous fluid was cultured in Sabouraud dextrose agar (SDA). Seven days later, white colonies formed and were identified as *Earliella scabrosa* via internal transcribed spacer (ITS) sequence analysis. In addition, the vitreous fluid was further cultured on an SGA incubator plate at 28 °C for 2 weeks. Figure 2a presents the white filamentous mold colonies growing on this plate. The hyphae stained positive for acridine orange, exhibiting an extremely thick cell wall, sparse septae, and internal nuclei (Fig. 2b). The timeline for the patient’s hospital course and treatment is presented in Fig. 3.

The isolates were identified by sequencing the ITS region as previously described [3]. Briefly, the genomic DNA was extracted from a mycelial mass using a Fungal Microbial DNA Isolation Kit (Solarbio Life Sciences Laboratories, Beijing, PRC) in accordance with the manufacturer’s instructions. For this test, 2 μl of fungal DNA and 12.5 μl of the Taq Master Mix (Vazyme Biotech Co., Ltd., Nanjing, PRC) with fungal specific primers were added to a 25-μl total reaction. The thermal cycling parameters were as follows: initial denaturation at 95 °C for 5 min; 35 cycles of denaturation at 95 °C for 30 s, annealing at 60 °C for 30 s, extension at 72 °C for 30 s; and a final extension at 72 °C for 10 min. The sequence of the amplified ITS PCR product that as obtained from the strain isolated in this study was analyzed using the National Center for Biological Information (NCBI) GenBank database and identified as *Earliella scabrosa* (KR706165, identity 99%). A sequence alignment revealed that this sequence shared 91% similarity with *Trametes* sp. and 89% similarity with *Ganoderma* sp.

## Discussion and conclusion

Most acute post-cataract endophthalmitis cases reported worldwide are caused by bacterial infections [4, 5]. In this case, the patient complained of decreased vision and red eye within 72 h postoperatively. The patient was then suspected to suffer from acute endophthalmitis induced by bacterial infection. Therefore, empirical treatment consisting of a vitrectomy followed by intraocular irrigation with vancomycin was administered. However, *Earliella scabrosa*, a type of fungus, was isolated from the vitreous fluid and identified by sequencing the ITS region. In retrospect, this case is not the only acute endophthalmitis case associated with fungi. Although fungal-associated, acute, post-cataract endophthalmitis is rare, dozens of cases have been reported in tropical regions [6]. *Aspergillus* spp., *Acremonium falciforme* and *Candida* spp. are included in the spectrum of etiological agents.

*Earliella scabrosa* is a genus of fungi named by Gilbertson and Ryvarden in 1985 that belongs to the family Polyporaceae [7]. *Earliella scabrosa* is considered a plant pathogen, exhibiting a strong association with freshwater forested wetlands in tropical areas, such as Micronesia [8]. Notably, a recent case report published by Desmond Shi-Wei Lim et al. was the first to document that this organism can infect humans [9]. In Lim’s report, this pathogen caused cutaneous fungal septic emboli in an immunocompromised child, resulting in mortality. However, data regarding human disease remains limited.

Here, we report a case in which a human eye infection was associated with *Earliella scabros*a. Several points should be noted in this report. First, *Earliella scabrosa* appears to be a novel opportunistic pathogen that can cause endophthalmitis after a cataract extraction. In this case, the patient had suffered from type II diabetes mellitus for 10 years. This disease can impair the immune response of a patient and increase the risk of infection after an operation [10]. Second, ITS sequence analysis is a reliable molecular method for fungal identification, especially for a rare pathogen [11]. As mentioned previously, data on *Earliella scabrosa* are limited; therefore, the pathogen isolated from the patient in this case was considered to be an unidentified contaminant, given the lack of morphological characteristics and an absence of sporulation. This fungus was finally identified by genomic level evidence. Third, endophthalmitis induced by *Earliella scabrosa* may have a favorable prognosis. IOL explantation with capsular bag removal and a 23G pars plana vitrectomy combined with silicone oil tamponade seemed to be an effective treatment. In this case, the patient’s vision post-operatively recovered from light perception to 20/200 without antifungal drug application. We noticed that there is considerable discrepancy between the prognoses described in Lim’s and this report. In Lim’s report, this pathogen resulted in death despite the use of massive antifungal treatment. However, in this case, the course was mild. One potential reason for this discrepancy is that the characteristics of the site of initial presentation were different. In the child described in Lim’s report, the skin was the initial site of infection. The vascular network within the skin may have facilitated the formation of vascular emboli by fungal hyphae, resulting in the dissemination of the infection and multi-organ involvement. On the contrary, the vitreous of the eye can, to some extent, restrict the dissemination of a pathogen because it contains no blood vessels and is a poor nutritional source for invading agents [12]. Another potential reason was that there were differences in the general physical condition of the two patients. The adult patient had type II diabetes but was in generally stable physical condition. However, the child described in Lim’s case was in poor physical condition during the course of admission. In addition to the fungal infection, the child had severe idiopathic aplastic anemia and was suffering from graft failure, intracranial hemorrhage and multiple bacterial infections. The child’s death may therefore have been the result of multiple diseases. Environmental risk factors were not assessed in our study because the surgery was performed in a local hospital before the patient was referred to Hainan Eye Hospital.

In conclusion, in this case, we reveal that *Earliella scabrosa*, a rare known fungal agent, was the underlying etiology in a human eye infection. These data reinforce the need to enhance awareness of fungal infections in acute endophthalmitis. Early diagnosis and prompt surgical treatment can improve the prognosis in affected patients.

## Abbreviations

IOL: Intraocular lens
ITS: Internal transcribed spacer
NCBI: National Center for Biological Information
SDA: Sabouraud dextrose agar
